# Experiences of Self-Collected Human Papilloma Virus (HPV) Testing Among Women Aged 20 to 65 Years in Ho Chi Minh City, Vietnam

**DOI:** 10.3390/diagnostics15080968

**Published:** 2025-04-10

**Authors:** Ai H. T. Pham, Thao H. Ha, Thanh Q. Le, Dat Q. Nguyen, Tuan M. Vo

**Affiliations:** 1 General Gynecology Department, TuDu Hospital, Ho Chi Minh City 7000, Vietnam; 2 OB-GYN Department, University of Medicine and Pharmacy at Ho Chi Minh City, Ho Chi Minh City 7000, Vietnam

**Keywords:** HPV sample self-collection, high-risk HPV, cervical cancer, probability proportional to size

## Abstract

**Background/Objectives:** To assess the correct sampling rates and self-collection satisfaction levels among female residents aged 25 to 64 years during first-time HPV testing in the communities of Ho Chi Minh City. **Methods:** An observational study was conducted on self-collection for HPV testing in communities from January to December 2024. The study employed a probability proportional to size sampling method, involving self-collected sampling and post-collection direct interviews. **Results:** The data show that 99.9% [95% CI = 0.99–1] of 775 women successfully collected their own samples during a first-time HPV testing process. The self-collection satisfaction rate was 80.4% [95% CI = 0.77–0.83]. **Conclusions:** Self-collected sampling for HPV testing has a very high success rate in communities. Moreover, women who feel confident in self-collection tend to have higher satisfaction rates with this new method. Therefore, self-collection sampling should be widely adopted for early cervical cancer screenings to test for high-risk HPV. It is essential to provide careful instructions and mobilization to encourage women’s confidence in performing self-collections.

## 1. Introduction

In 2020, cervical cancer ranked as the eighth leading cancer type in Vietnamese women, according to statistics from the International Agency for Research on Cancer (IARC) [1]. This ranking highlights the success of early screening and treatment programs for precancerous lesions.

High-risk strains of the human papillomavirus (HPV) cause most cervical cancer cases [2,3]. Although most individuals are clear of infection within two years, long-term exposure can lead to precancerous lesions and cervical cancer if left undiagnosed and untreated [4]. Highly sensitive high-risk HPV tests increase the likelihood of detecting precancerous lesions in the cervix compared to cytology testing [5,6,7]. Current cervical cancer screening programs primarily focus on HPV testing, with organizations such as the American College of Obstetricians and Gynecologists (ACOG), the American Cancer Society (ACS), and the American Society for Colposcopy and Cervical Pathology (ASCCP) in the United States recommending HPV tests as the first line of screening for women aged 25 and above [8,9,10].

Samplers for HPV self-collection have been implemented in national screening programs worldwide [11]. The World Health Organization (WHO) recommended self-collection as a tool for cervical cancer screening globally in 2022 [2,12]. This method can effectively increase participation in global cervical cancer screening programs, with the potential to enhance screening coverage by up to 97% [13,14].

Ho Chi Minh City (HCMC) is the most populous city in Vietnam and a crucial hub for the country’s economic, cultural, and educational development. Due to its high population density, community healthcare, particularly women’s health, is a top priority in the city’s social policies.

Vietnam has approved the use of human papillomavirus (HPV) tests as a cervical cancer screening method. Since 2016, HPV testing has been the primary screening tool for women aged 30 and above [15]. We are in a transition period from a less developed country to a developed country, and most people do not have enough time or money to go to the hospital to test for high-risk HPV. The use of self-sampling in HPV testing will correct the above and help increase the rate of people being screened for early cervical cancer. Furthermore, to combat and eventually eradicate cervical cancer, Vietnam’s Ministry of Health has approved self-collection HPV tests as a cervical cancer screening tool since 2021 [16].

There is currently a lack of data on self-collection HPV testing in Vietnamese communities, particularly in HCMC. Therefore, a study was conducted to investigate the rate of valid samples obtained through self-collection HPV testing among women aged 25–65 from HCMC communities and their satisfaction with the sample self-collection process. The study aims to investigate the effectiveness of self-collection HPV testing in providing valid samples among women from HCMC communities, aged 25–65 years old, and to evaluate their satisfaction with the self-collection process.

## 2. Methods

### 2.1. Study Design and Participants

The study employed a non-participant observation method to collect data on self-collection human papillomavirus (HPV) testing from January to December 2024.

The study included female residents of HCMC who were between 25–65 years old, had participated in sexual activity, and were free from any vaginal issues within the past 24 h before sample collection. The participants did not have a history of cervical cancer, precancerous lesions, or abnormal cell readings from regular health check-ups. The study excluded women who were incapable of giving informed consent due to mental or cognitive impairments.

### 2.2. Sample Size

A formula was used to estimate with high precision the largest sample size in the population when the estimated prevalence is 50%. Because this prevalence fluctuates significantly, we choose *p* = 0.5 to ensure the best sample power for the main objective of the study. As the study focused on community settings, the sample size was doubled to mitigate design effects, resulting in a minimum sample size of 772 women to ensure sufficient power.

### 2.3. Data Collection Tools

Two tools were utilized, a pre-composed questionnaire administered before testing and a test result recorder following HPV sample self-collection. The questionnaire consisted of two sections, with Section 1 containing 36 questions that covered epidemiology, medical history, and knowledge about HPV testing prior to self-collection. Section 2 included 57 questions that explored women’s experiences and practices during self-collection steps and post-collection experiences.

“*Self-collection samples*”: The Medic Hoahao Hospital’s Department of Medical Genetics Testing notified the research team on the test sample’s compliance with the required criteria. Non-compliant samples were noted on a recorder. In cases where the self-collected sample was deemed invalid, a message was conveyed indicating the need for re-collection, citing “insufficient cells”.

“*To gauge satisfaction*”: the research team utilized a specific variable, which was defined by a single question querying the individual’s preference for self-collection during the next testing process. A positive response to this question, which favored self-collection, was used to determine the level of satisfaction.

### 2.4. Study Procedure

Step 1: Sampling.

**Figure 1:**
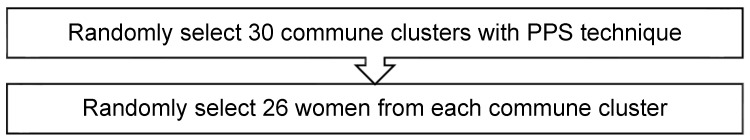
Sampling process.

The sampling process began with selecting clusters using the probability proportional to size (PPS) technique, as depicted in Figure 1. The definition of a cluster in the study was a commune.

Initially, 30 clusters of communes were randomly chosen from 312 communes in HCMC. Women participants were then selected from a list of 4,764,900 women provided by the Department of Population and Family Planning, which is under the management of the HCMC Department of Health. Each cluster was assigned a specific number of women to be selected, with the first cluster consisting of women with a random number of 68. Subsequent clusters included women with numbers 68 + 158,830, with subsequent numbers calculated by adding the product of (*n* − 1) and 158,830 to 68.

At the second stage, 26 women were randomly chosen from each selected commune. A total of 780 women were recruited into the study between January and December 2024. The percentage of women who provided informed consent was 99.4%, with 775 women participating in the study.

Step 2: Pilot study.

Ten women were randomly selected and interviewed to refine a questionnaire, ensuring it aligned with local customs and daily language. These 10 women were not part of the main study participant group.

Step 3: Data collection with interview questionnaire

We contacted the heads of the communal health station to gather information on the local area and finalize the plan and schedule for collecting test samples. We planned to send our research team to participant houses on Saturdays and Sundays, twice a day, from 8:00 to 11:30 in the morning and 15:00 to 19:00 in the afternoon, to collect test samples and gather related information alongside the health station staff.

**Figure 2:**
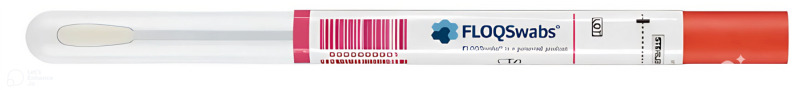
Cotton FLOQSwabs for self-collection.

To ensure a smooth self-collection process, we provided participants with illustrated instructions for the research subject at home on how to collect samples using cotton FLOQSwabs. These swabs are self-sampling kits from Roche^R^ (Basel, Switzerland) (Figure 2). As soon as a participant provided the test sample, our team diluted it into a solvent liquid and stored it according to the recommended procedure to maintain the sample’s quality and minimize solvent waste. Each vial contains 20 mL methanol solution. To avoid errors during the collection process, the sample was then labeled with a study code. After collecting, the samples were stored in specialized containers, as specified by the manufacturer.

Upon completing the self-collection process, a collaborator conducted an interview with the participant using a pre-designed questionnaire to record their experiences. The interviews took approximately 5–10 min. To ensure accuracy, a midwife verified the test code on the participant’s record with the corresponding code on the test order sheet before transporting the sample to the Department of Medical Genetics at Medic Hoahao Hospital on the same day. The department received the samples within 24 h and analyzed them using the Roche Cobas Z480 system (Basel, Switzerland), which specializes in HPV analysis, in accordance with the hospital’s testing procedure. Once the test results were available, we contacted the participants to inform them of the outcome.

Step 4: Counseling and patient instructions

In cases where the initial self-collected test sample was deemed invalid, we reached out to the participant to advise on re-collection procedures. Upon confirmation of a positive result of high-risk HPV, we provided participants with the necessary health care guidance in accordance with MOH (Ministry of Health) recommendations.

### 2.5. Data Analysis

The data analysis was performed using STATA 17.0 software. The mean and standard deviation of continuous variables, as well as rates and percentages of categorical variables, were calculated and presented. To investigate the connection between self-collection satisfaction and other factors, univariate and multivariate logistic regression analyses were conducted. Prevalence odds ratios and corresponding 95% confidence intervals were calculated and documented.

### 2.6. Ethical Considerations

This study was conducted in accordance with the Declaration of Helsinki. Ethics approval for the study was obtained from the HCMC University of Medicine and Pharmacy Ethics Council on Bio-Medical Research via Decision No. 1257/HDDD-DHYD issued on 14 December 2023. Additionally, agreements were obtained from relevant District Health Centers and Communal Health Stations. Written informed consent for participation was obtained from all subjects involved in the study. Data was kept anonymous and confidential during all stages of the study.

## 3. Results

A total of 780 women were invited to participate in a self-sampling research project, with 775 ultimately completing the study. Five individuals opted out, stating that they were preoccupied with domestic duties as the reason for their non-participation. Given the very low decline rate of less than 1%, the group that declined to participate was not subject to further analysis.

The characteristics of the study’s participants are given in Table 1. The average age of the participants was 46 ± 10 years, ranging from 25 to 65 years. Most participants were over the age of 35, accounting for approximately 82.2%. Few participants were unmarried, comprising only 1.7% of the group. Most participants had established their own families. The mean age at which participants were married was 25 ± 5 years, ranging from 16 to 58 years.

Table 2 shows the rates of successful self-collected samples and post-collection satisfaction for HPV tests. The vast majority, 99.9% (95% CI: 99.6–100.0), of self-collected samples were valid at the initial collection. Most women, 80.4% (95% CI: 77.6–83.2), reported being satisfied with the self-collection process.

After univariate analysis with 31 variable pairs, we noted 10 related factors with statistical significance for POR of post-collection satisfaction, including occupation, educational level, average income, completion of eight self-collection steps, vulvo-vaginal burning sensation, vulvar pain, vaginal pain, confidence in correct self-collection, feelings regarding self-collection duration, and feelings regarding the process of self-collection. We combined the ten above-mentioned independent variables with two potential variables (lower abdominal discomfort and pain during urination, *p* ≤ 0.2) into multivariate regression equations to control for confounders and co-impact factors. Table 3 summarizes the 12 variables in the multivariate analysis.

After analysis with multivariate regression model, it was found that those who were confident in successful self-collection performance at the first collection had an adjusted POR for post-collection satisfaction of 2.68 times (95% CI: 1.51–4.75) higher than those without confidence (*p* < 0.05). Participants who felt shy or very shy about performing self-collection had an adjusted POR for post-collection satisfaction of 0.34 times (95% CI: 0.13–0.87) higher than those without shyness. In addition, after multivariate analysis, the relationship between post-collection satisfaction and other related factors (occupation, educational level, average income, completion of eight self-collection steps, vulvar or vaginal burning sensation, pain in vulva, pain in vagina, feelings regarding self-collection duration, lower abdominal discomfort, and pain during urination) turned out to be not statistically significant (adjusted *p* > 0.05).

## 4. Discussion

This study involving 775 women in HCMC communities found that 99.9% (774/775) achieved successful sample self-collection for HPV testing, with only one failure (0.1%)

As compared with other international studies (Table 4), we found that successful test sampling in our study has some differences. In the study by Bais et al. [17] conducted in The Netherlands in 2007, the sample size was equivalent to ours, but the self-collection failure was higher. It is possible that their self-collected samples were stored in solvent and transported by postal services, then processed by centrifuge at a laboratory while we ensured that sample transportation to laboratory occurred within 24 h after the samples were taken. Another study by Gok [18] in The Netherlands was conducted in 2010 with a larger sample size (7404 women), in which the failed sample rate was 0.3%—very similar to ours. Darlin et al. [19] recorded a rate of failed test samples of 1.4%, which was higher than ours, and this discrepancy might be the result of self-collection locations. The collections were carried out at a clinic in the Darlin study, while in our study, they took place in the participants’ homes, and participant shyness might affect the self-collection. Modibbo [20] and Brewer [21] recorded failed sample rates at 2.7% and 1.0%, respectively, which were similar to that of Bais et al. [17], and they did not control sample storage time. The length of time for sample storage may affect the quality of the self-collected samples. For instance, in the study by Gizaw et al. [22], the failed sample rate reached 19.2%; such a high failure rate might be due to a sample storage time after self-collection of up to 7 days. In general, failed sample rates after self-collection for HPV testing appeared very low, but more studies are needed to assess the effect of sample storage time after self-collection.

Our study found that post-collection satisfaction was 80.4% (95% CI: 77.6–83.2). Although there are not many studies in the world that examine participant experience during self-collection, in Enerly’s study [23], where 267 women underwent HPV screening either by sample self-collection or by medical staff, the former was preferred, with 63.3% choosing self-collection. In the study by Tranberg [24], among 1242 women who underwent cervical cancer screening, 51.1% selected self-collection HPV tests while 48.9% opted for cytology. A rare study by Winer [25] reported that among 1206 women who underwent self-collection for HPV testing, 91.0% accepted self-collection for repeated HPV tests. In general, the self-collection acceptance rate in women was high and was even higher if they had already experienced this method.

The factors influencing self-collection for future HPV testing included confidence in correct manipulation and shyness during first performance. This shows that the first self-collection experience will affect a woman’s decision regarding preference for future testing methods.

New applicability: Our research is a pioneering effort to evaluate the suitability of self-collected samples for HPV testing among community women in Ho Chi Minh City and Vietnam. This study aimed to gather data on the success rate and participant satisfaction with the self-collection method for HPV testing.

Limitations of the study: The storage and administrative process duration for samples self-collected by participants was 24 h, with medical staff collecting the samples and transporting them to the lab within this timeframe. However, if participants sent their samples via postal services, this timeline could be extended, potentially compromising sample quality. Several studies have demonstrated the reliability of dry self-collected swabs for a time period of several days [26]. This statement may raise questions about the application of this tool during screening.

## 5. Conclusions

We observed a high success rate for first-time HPV testing using self-collected samples, marking a significant milestone in community-based HPV testing studies in Ho Chi Minh City. Women who demonstrated confidence in the self-collection process reported higher satisfaction levels with this method. Implementing early cervical cancer screening through the self-collection method for high-risk HPV testing in communities could be an effective approach. To ensure the success of this approach, it is essential to provide women with thorough guidance and mobilize them to build their confidence regarding the self-collection process.

## Figures and Tables

**Table 1:** Characteristics of study participants (*n* = 775).

Description	Number (*n* = 775)	Percent (%)
Age *: 46 ± 10 [25–65]		
≤35 yrs	138	17.8
>35 yrs	637	82.2
**Occupation**		
Housework	328	42.3
Manual labor	43	5.6
Intellectual labor	299	38.6
Business	105	13.6
**Marital status**		
Currently married	701	90.5
Divorced/separated/widowed	61	7.9
Unmarried	13	1.7
Age of marriage * (*n* = 762): 25 ± 5 [16–58]		

*: Mean ± standard deviation [min–max values].

**Table 2:** Valid samples by self-collection for HPV tests and post-collection satisfaction (n = 775).

Description	Number (*n* = 775)	Percent (%)	95% CI
**Sample at first self-collection**			
Valid	774	99.9	99.6–100.0
Invalid	1	0.1	0.0–0.4
**Satisfaction**			
Yes	623	80.4	77.6–83.2
No	152	19.6	16.8–22.4

**Table 3:** Relationship between self-collection satisfaction and related factors.

Characteristics	Self-Collection Satisfaction		POR	Adjusted POR	95% CI	Adjusted *p*
	Yes *n* = 623 (%)	No *n* = 152 (%)				
**Occupation**						
Manual labor	37 (86.0)	6 (14.0)	1.89	1.54	0.61–3.89	0.36
Intellectual labor	250 (83.6)	49 (16.4)	1.57	1.20	0.67–2.14	0.54
Business	85 (81.0)	20 (19.0)	1.30	1.29	0.72–2.32	0.39
Housework	251 (76.5)	77 (23.5)	1	1		
**Educational level**						
High school	158 (80.6)	38 (19.4)	1.36	1.36	0.84–2.23	0.21
Higher education	281 (83.9)	54 (16.1)	1.70	1.31	0.72–2.40	0.37
Lower school	184 (75.4)	60 (24.6)	1	1		
**Average income**						
Low (<VND5 mil)	145 (73.6)	52 (26.4)	0.56	0.69	0.43–1.12	0.14
High (VND10–20 mil)	121 (80.7)	29 (19.3)	0.83	0.81	0.48–1.34	0.40
Very high (>VND20 mil)	33 (84.6)	6 (15.4)	1.10	0.89	0.35–2.28	0.81
Medium (VND5–10 mil)	324 (83.3)	65 (16.7)	1	1		
**Completion of 8 self-collection steps**						
Yes	589 (81.6)	133 (18.4)	2.47	1.81	0.95–3.48	0.07
No	34 (64.2)	19 (35.8)	1	1		
**Vulvo-vaginal burning**						
Yes	60 (71.4)	24 (28.6)	0.57	0.86	0.48–1.56	0.63
No	563 (81.5)	128 (18.5)	1	1		
**Lower abdominal discomfort**						
Yes	7 (63.6)	4 (36.4)	0.42	0.61	0.15–2.43	0.49
No	616 (80.6)	148 (19.4)	1	1		
**Vulvar pain**						
Yes	10 (58.8)	7 (41.2)	0.34	0.63	0.15–2.60	0.52
No	613 (80.9)	145 (19.1)	1	1		
**Vaginal pain**						
Yes	19 (57.6)	14 (42.4)	0.31	0.64	0.24–1.65	0.35
No	604 (81.4)	138 (18.6)	1	1		
**Pain during urination**						
Yes	10 (66.7)	5 (33.3)	0.48	1.24	0.29–5.38	0.77
No	613 (80.7)	147 (19.3)	1	1		
**Confidence in correct collection**						
Yes	584 (82.5)	124 (17.5)	3.38	2.68	1.51–4.75	**<0.01**
No	39 (58.2)	28 (41.8)	1	1		
**Feelings regarding collection duration**						
Long	210 (75.8)	67 (24.2)	0.65	0.77	0.53–1.14	0.19
Not long	413 (82.9)	85 (17.1)	1	1		
**Feelings regarding self-collection**						
Shy–very shy	613 (81.2)	142 (18.8)	0.23	0.34	0.13–0.87	**0.02**
Not shy	10 (50.0)	10 (50.0)	1	1		

Bold values mean statistical significance. Adjusted values by multivariate regression model. POR: proportional odds ratio.

**Table 4:** Comparison of failed self-collected samples from various studies.

Authors	Country	Sample Size	Percent of Failed Samples (%)
Bais [17] (2007)	The Netherlands	736	1.2
Gok [18] (2010)	The Netherlands	7404	0.3
Darlin [19] (2013)	Sweden	147	1.4
Modibbo [20] (2017)	Nigeria	185	2.7
Gizaw [22] (2019)	Ethiopia	721	19.2
Brewer [21] (2021)	New Zealand	305	1.0
Our study (2024)	Vietnam	775	0.1

## Data Availability

The original contributions presented in this study are included in the article. Further inquiries can be directed to the corresponding author.

## References

[1] WHO (2022). All Cancers. https://gco.iarc.who.int/media/globocan/factsheets/cancers/39-all-cancers-fact-sheet.pdf.

[2] WHO (2021). WHO Guideline for Screening and Treatment of Cervical Pre-Cancer Lesions for Cervical Cancer Prevention, Second Edition. https://www.who.int/publications/i/item/9789240030824.

[3] Okunade K.S. (2020). Human papillomavirus and cervical cancer. J. Obstet. Gynaecol..

[4] Chan C.K., Aimagambetova G., Ukybassova T., Kongrtay K., Azizan A. (2019). Infection and Cervical Cancer: Epidemiology, Screening, and Vaccination-Review of Current Perspectives. J. Oncol..

[5] Phoolcharoen N., Kantathavorn N., Krisorakun W., Taepisitpong C., Krongthong W., Saeloo S. (2018). Acceptability of Self-Sample Human Papillomavirus Testing Among Thai Women Visiting a Colposcopy Clinic. J. Community Health.

[6] Gupta S., Palmer C., Bik E.M., Cardenas J.P., Nuñez H., Kraal L., Bird S.W., Bowers J., Smith A., Walton N.A. (2018). Self-Sampling for Human Papillomavirus Testing: Increased Cervical Cancer Screening Participation and Incorporation in International Screening Programs. Front. Public Health.

[7] Cuzick J., Adcock R., Kinney W., Castle P.E., Robertson M., McDonald R.M., Stoler M.H., Du R., Wheeler C.M., New Mexico HPV Pap Registry Steering Committee (2023). Impact of HPV testing in opportunistic cervical screening: Support for primary HPV screening in the United States. Int. J. Cancer.

[8] The American College of Obstetricians and Gynecologists (ACOG) (2023). Updated Cervical Cancer Screening Guidelines. https://www.acog.org/clinical/clinical-guidance/practice-advisory/articles/2021/04/updated-cervical-cancer-screening-guidelines.

[9] Fontham E.T.H., Wolf A.M.D., Church T.R., Etzioni R., Flowers C.R., Herzig A., Etzioni E.R., Flowers C.R., Herzig A., Guerra C.E. (2020). Cervical cancer screening for individuals at average risk: 2020 guideline update from the American Cancer Society. CA Cancer J. Clin..

[10] Perkins R.B., Guido R.S., Castle P.E., Chelmow D., Einstein M.H., Garcia F., Huh W.K., Kim J.J., Moscicki A.-B., Nayar R. (2020). 2019 ASCCP Risk-Based Management Consensus Guidelines for Abnormal Cervical Cancer Screening Tests and Cancer Precursors. J. Low Genit. Tract. Dis..

[11] Serrano B., Ibáñez R., Robles C., Peremiquel-Trillas P., de Sanjosé S., Bruni L. (2022). Worldwide use of HPV self-sampling for cervical cancer screening. Prev. Med..

[12] WHO (2020). WHO Cervical Cancer Elimination Intiative: From Call to Action to Global Movement. https://www.who.int/publications/m/item/who-cervical-cancer-elimination-initiative--from-call-to-action-to-global-movement.

[13] Onuma T., Kurokawa T., Shinagawa A., Chino Y., Yoshida Y. (2020). Evaluation of the concordance in HPV type between self- and physician-collected samples using a brush-based device and a PCR-based HPV DNA test in Japanese referred patients with abnormal cytology or HPV infection. Int. J. Clin. Oncol..

[14] Blomberg K., Hälleberg-Nyman M. (2023). Experiences of human papillomavirus self-sampling by women > 60 years old: A qualitative study. Health Expect..

[15] Vietnam Ministry of Health (2016). The National Action Plan on Cervical Cancer Control for the period of 2016–2025. https://thuvienphapluat.vn/van-ban/The-thao-Y-te/Quyet-dinh-5240-QD-BYT-2016-Hanh-dong-quoc-gia-du-phong-va-kiem-soat-ung-thu-co-tu-cung-430440.aspx.

[16] Vietnam Ministry of Health (2021). Additional Guidelines on Early Screening and Management of Breast and Cervical Cancers in Communities by Project No. 818 until 2030. Decison No. 1639/QĐ-BYT Promulgated on 19/3/2021.

[17] Bais A.G., van Kemenade F.J., Berkhof J., Verheijen R.H., Snijders P.J., Voorhorst F., Babović M., van Ballegooijen M., Helmerhorst T.J., Meijer C.J. (2007). Human papillomavirus testing on self-sampled cervicovaginal brushes: An effective alternative to protect nonresponders in cervical screening programs. Int. J. Cancer.

[18] Gök M., Heideman D.A.M., van Kemenade F.J., Berkhof J., Rozendaal L., Spruyt J.W.M., Voorhorst F., Beliën J.A.M., Babović M., Snijders P.J.F. (2010). HPV testing on self collected cervicovaginal lavage specimens as screening method for women who do not attend cervical screening: Cohort study. BMJ.

[19] Darlin L., Borgfeldt C., Forslund O., Hénic E., Hortlund M., Dillner J., Kannisto P. (2013). Comparison of use of vaginal HPV self-sampling and offering flexible appointments as strategies to reach long-term non-attending women in organized cervical screening. J. Clin. Virol..

[20] Modibbo F., Iregbu K.C., Okuma J., Leeman A., Kasius A., de Koning M., Quint W., Adebamowo C. (2017). Randomized trial evaluating self-sampling for HPV DNA based tests for cervical cancer screening in Nigeria. Infect. Agents Cancer.

[21] Brewer N., Bartholomew K., Grant J., Maxwell A., McPherson G., Wihongi H., Bromhead C., Scott N., Crengle S., Foliaki S. (2021). Acceptability of human papillomavirus (HPV) self-sampling among never- and under-screened Indigenous and other minority women: A randomised three-arm community trial in Aotearoa New Zealand. Lancet Reg. Health West Pac..

[22] Gizaw M., Teka B., Ruddies F., Abebe T., Kaufmann A.M., Worku A., Wienke A., Jemal A., Addissie A., Kantelhardt E.J. (2019). Uptake of Cervical Cancer Screening in Ethiopia by Self-Sampling HPV DNA Compared to Visual Inspection with Acetic Acid: A Cluster Randomized Trial. Cancer Prev. Res..

[23] Enerly E., Bonde J., Schee K., Pedersen H., Lönnberg S., Nygård M. (2016). Self-Sampling for Human Papillomavirus Testing among Non-Attenders Increases Attendance to the Norwegian Cervical Cancer Screening Programme. PLoS ONE.

[24] Tranberg M., Bech B.H., Blaakær J., Jensen J.S., Svanholm H., Andersen B. (2018). Preventing cervical cancer using HPV self-sampling: Direct mailing of test-kits increases screening participation more than timely opt-in procedures—A randomized controlled trial. BMC Cancer.

[25] Winer R.L., Lin J., Tiro J.A., Miglioretti D.L., Beatty T., Gao H., Kimbel K., Thayer C., Buist D.S.M. (2019). Effect of Mailed Human Papillomavirus Test Kits vs. Usual Care Reminders on Cervical Cancer Screening Uptake, Precancer Detection, and Treatment: A Randomized Clinical Trial. JAMA Netw. Open.

[26] Sechi I., Muresu N., Puci M.V., Saderi L., Del Rio A., Cossu A., Muroni M.R., Castriciano S., Martinelli M., Cocuzza C.E. (2023). Preliminary Results of Feasibility and Acceptability of Self-Collection for Cervical Screening in Italian Women. Pathogens.

